# An increase in hookworm infection temporally associated with ecologic change.

**DOI:** 10.3201/eid0303.970321

**Published:** 1997

**Authors:** B Lilley, P Lammie, J Dickerson, M Eberhard

**Affiliations:** University of Alabama at Birmingham, Birmingham, Alabama, USA.

## Abstract

This report describes a significant increase in the prevalence of hookworm infection in an area of Haiti where intestinal parasites are common, but hookworm has not been common. Changing environmental conditions, specifically deforestation and subsequent silting of a local river, have caused periodic flooding with deposition of a layer of sandy loam topsoil and increased soil moisture. We speculate that these conditions, conducive to transmission of the infection, have allowed hookworm to reemerge as an important human pathogen.


### An Increase in Hookworm Infection Temporally Associated With Ecologic Change

This report describes a significant increase in the prevalence of hookworm infection in an area of Haiti where intestinal parasites are common, but hookworm has not been common. Changing environmental conditions, specifically deforestation and subsequent silting of a local river, have caused periodic flooding with deposition of a layer of sandy loam topsoil and increased soil moisture. We speculate that these conditions, conducive to transmission of the infection, have allowed hookworm to reemerge as an important human pathogen.

Infectious disease patterns in human populations are influenced by changes in human behavior, socioeconomic conditions, and environmental factors. Technologic advances can bring about global changes in climate and disease patterns, often with adverse effects (1,2). Shifts in land and water use as a consequence of economic development or environmental degradation may increase contact between disease agents and human populations (3). Environmental changes may directly increase exposure to infectious agents or indirectly increase disease transmission by expanding vector habitat. Altered or fluctuating ecologic conditions have resulted in a marked increase in infection rates and associated diseases for many infectious organisms, including parasites (1,2,4). As an example, the marked changes in schistosomiasis patterns in Egypt have been attributed to the construction of the Aswan High Dam (5,6). More clearly, in the Senegal River basin, construction of irrigation canals and dams and a rapid influx of workers to the area resulted in an unprecedented increase of schistosomiasis from 0% to >95% over a 3-year period (7). Changing ecologic conditions have also been responsible for marked increases in malaria transmission and drastic shifts in anopheline vector populations (2,8). In Brazil, for example, deforestation and migration of people into the interior have resulted in a fivefold increase in malaria prevalence and a shift from *Plasmodium vivax* to *P. falciparum* as the predominant species (9,10). Further evidence of the effect of climatic changes on infectious disease patterns is seen in outbreaks of malaria after floods or heavy monsoon rains associated with El Niño-Southern oscillations (11-13).

Typically, intestinal helminth infections would not be considered new or reemerging as they are highly prevalent throughout the developing world. Infection levels tend to be stable because of the lack of sanitary waste disposal and proper hygiene practices (14). However, under appropriate conditions, geohelminth infections can reemerge in areas of low prevalence. We report here a rapid increase in hookworm prevalence that coincided with ecologic change from political turmoil in the Haitian community of Leogane. The increase may be an indirect consequence of deforestation, which led to silt accumulation in the local river, subsequent flooding, altered water drainage patterns, and saturation of soil near homes.

Leogane is a small, urban community surrounded by extensive sugar cane fields (15). Access to piped water is limited, and there is no system for sewage disposal. Most people live in mud or concrete block dwellings with no electricity or indoor plumbing and few latrines. Children are exposed early in life to many parasites, including intestinal helminths. Peak prevalence of *Ascaris lumbricoides* and *Trichuris trichiura*, as well as other parasites, occurs by 24 to 30 months of age (data not shown).

Data were collected in an ongoing longitudinal study of risk factors for Bancroftian filariasis in children. Open enrollment of children under 2 years of age from several neighborhoods in Leogane took place in 1990 (approximately 60 children) and continued through 1996 (more than 250 children). Stool samples were collected at approximately 6- to 9-month intervals and were examined by the Formalin-ethyl acetate concentration technique (16). Children found infected with helminths were treated with mebendazole.

Reinfection was common, necessitating retreatment. No other systematic treatment or deworming programs were ongoing in Leogane during this period.

Throughout the 6-year follow-up, the prevalence of *Ascaris* and *Trichuris* remained relatively stable (Figure). In contrast, the prevalence of hookworm infection increased markedly from 0% to 12%-15% over the 6-year period; most of this increase took place in 3 years, beginning in 1993 through 1995.

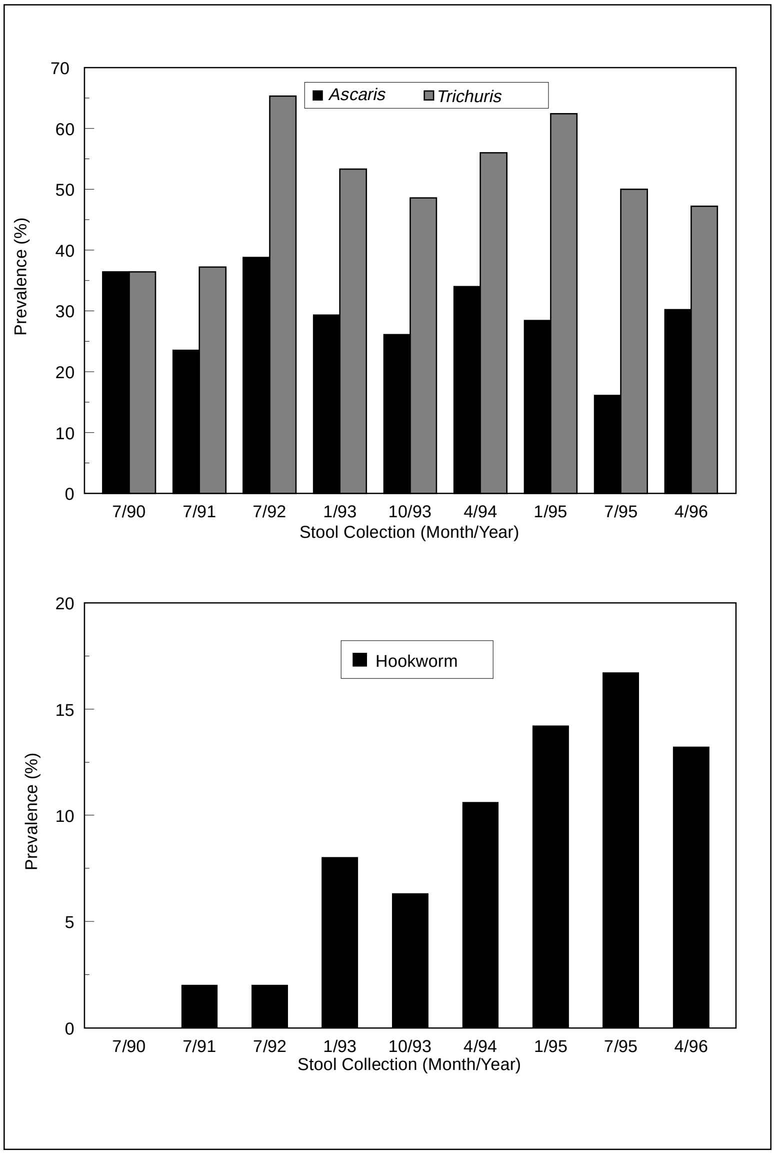

Figure. Upper panel: The prevalence of *Ascaris* (solid bars) and *Trichuris* (hatched bars) for each of the indicated stool collection periods. Lower panel: The prevalence of hookworm infection for the same collection periods. A total of 881 stools were examined after Formalin-ethyl acetate concentration (mean 98 per collection period, range 33-174).

We postulate that changes in local environmental conditions favored hookworm transmission and allowed it to increase rapidly. Hookworm transmission is favored by moist conditions that allow optimum development and survival of the larvae. Hookworms differ from other geohelminths such as *Ascaris* and *Trichuris* in which the infective eggs are more desiccation-resistant than the infective larvae. The sandy loam soil deposited by the flooding may also have been more conducive to hookworm development and survival, thus heightening the effects of the increased soil moisture.

Conditions that favored the increase in hookworm prevalence may have occurred as a result of continued flooding of the River Royone, which runs south through the town of Leogane. Because of extensive deforestation of this river's watershed, the channel accumulates silt rapidly and must be maintained by heavy equipment. After political upheaval in 1990, maintenance of the river channel and its tributaries was no longer possible and beginning in 1992, the river frequently flooded Leogane and surrounding areas. Altered river drainage patterns turned much of the community into a delta; possibly changed the soil type, especially the surface deposits; and allowed the soil to remain moist and conducive to hookworm transmission. Thus, the increase in hookworm prevalence coincides temporally with the lack of maintenance of the river channel. We noted no other dramatic changes in rainfall, temperature, or other environmental conditions during this period. Additionally, although the cohort aged over this period, the average age of the children in the survey increased only from 21 to 44 months because of continuous enrollment of younger children. Thus, increases in hookworm prevalence are not likely to be age-related.

In summary, our observations illustrate the value of longitudinal surveillance data for monitoring disease prevalence. They further highlight the effects that environmental changes (the result of planned human activity or as in the present case, the indirect consequence of political strife) may have in shifts in prevalence of infectious diseases, including intestinal helminth infections.

**Bruce Lilley,* Patrick Lammie,† Jennifer Dickerson,† and Mark Eberhard†**
*University of Alabama at Birmingham, Birmingham, Alabama, USA; and
†Centers for Disease Control and Prevention, Atlanta, Georgia, USA

### References

1. Wilson M. Infectious diseases: an ecological perspective. BMJ 1995;311:1681-4.
2. Patz JA, Epstein PR, Burke TA, Balbus JM. Global climate change and emerging infectious diseases. JAMA 1996;275:217-23.
3. Stanley NF, Alpers MP, editors. Man-made lakes and human health. New York: Academic Press, 1975.
4. Jackson EK. Climate change and global infectious disease threats. Med J Aust 1995;163:570-4.
5. El Alamy MA, Cline BL. Prevalence and intensity of *Schistosoma haematobium* and *S. mansoni* infection in Qalyub, Egypt. Am J Trop Med Hyg 1997;26:470-2.
6. Abdel-Wahab MF. Schistosomiasis in Egypt. Boca Raton: CRC Press, 1982.
7. Gryseels B, Stelma FF, Talla I, van Dam GJ, Polman K, Sow S, et al. Epidemiology, immunology and chemotherapy of *Schistosoma mansoni* infections in a recently exposed community in Senegal. Trop Geogr Med 1994;46:209-19.
8. Coluzzi M. Malaria and the Afrotropical ecosystems: impact of man-made environmental changes. Parassitologia 1994;36:223-7.
9. McGreevy PB, Dietze R, Prata A, Hembree SC. Effects of immigration on the prevalence of malaria in rural areas of the Amazon basin of Brazil. Mem Inst Oswaldo Cruz 1989;84:485-91.
10. Sawyer D. Economic and social consequences of malaria in new colonization projects in Brazil. Soc Sci Med 1993;37:1131-6.
11. Mathur KH, Harpalani G, Kalra NL, Murthy GGK, Narasimham MVVL. Epidemic of malaria in Barmer District (Thar Desert) of Rajasthan during 1990. Indian J Malariol 1992;29:1-10.
12. Bouma MJ, Sondrop HE, van der Kay HJ. Climate change and periodic epidemic malaria. Lancet 1994;343:1440.
13. Bouma MJ, van der Kay HJ. Epidemic malaria in India and the El Nino southern oscillation. Lancet 1994;344:1638-9.
14. World Health Organization. Prevention and control of intestinal parasitic infections. World Health Organ Tech Rep Ser 1987;749:8.
15. Raccurt C, Lowrie RC, Katz SP, Duverseau YT. Epidemiology of *Wuchereria bancrofti* in Leogane, Haiti. Trans R Soc Trop Med Hyg 1988;82:721-5.
16. Markell EK, Voge M, John DT. Medical parasitology, Philadelphia: W.B. Saunders, 1992.
